# A genomic instability-associated lncRNA signature for predicting prognosis and biomarkers in lung adenocarcinoma

**DOI:** 10.1038/s41598-024-65327-3

**Published:** 2024-06-24

**Authors:** Chunxuan Lin, Kunpeng Lin, Pan Li, Hai Yuan, Xiaochun Lin, Yong Dai, Yingying Zhang, Zhijun Xie, Taisheng Liu, Chenggong Wei

**Affiliations:** 1 Department of Respiratory Medicine, Guangdong Provincial Hospital of Integrated Traditional Chinese and Western Medicine, Foshan, Guangdong 528200 People’s Republic of China; 2 Department of Abdominal Oncosurgery, Affiliated Cancer Hospital & Institute of Guangzhou Medical University, Guangzhou, Guangdong People’s Republic of China; 3 Affiliated Cancer Hospital & Institute of Guangzhou Medical University, Guangzhou, Guangdong People’s Republic of China; 4 Department of Cardio-Thoracic Surgery, Guangzhou Hospital of Integrated Chinese and Western Medicine, Guangzhou, Guangdong People’s Republic of China; 5 Department of Medical Examination Center, Guangzhou First People’s Hospital, School of Medicine, South China University of Technology, Guangzhou, Guangdong People’s Republic of China; 6 Department of Thoracic Surgery, Affiliated Cancer Hospital & Institute of Guangzhou Medical University, Guangzhou, Guangdong 510095 People’s Republic of China; 7 Department of Radiology Department, The Second People’s Hospital of Jiangmen, Jiangmen, Guangdong People’s Republic of China

**Keywords:** Lung adenocarcinoma, Genomic instability, lncRNA, Overall survival, Cancer, Computational biology and bioinformatics

## Abstract

Genomic instability (GI) was associated with tumorigenesis. However, GI-related lncRNA signature (GILncSig) in lung adenocarcinoma (LUAD) is still unknown. In this study, the lncRNA expression data, somatic mutation information and clinical survival information of LUAD were downloaded from The Cancer Genome Atlas (TCGA) and performed differential analysis. Functional and prognosis analysis revealed that multiple GI-related pathways were enriched. By using univariate and multivariate Cox regression analysis, 5 GI-associated lncRNAs (AC012085.2, FAM83A-AS1, MIR223HG, MIR193BHG, LINC01116) were identified and used to construct a GILncSig model. Mutation burden analysis indicated that the high-risk GI group had much higher somatic mutation count and the risk score constructed by the 5 GI-associated lncRNAs was an independent predictor for overall survival (OS) (P < 0.05). Overall, our study provides valuable insights into the involvement of GI-associated lncRNAs in LUAD and highlights their potential as therapeutic targets.

## Introduction

Lung cancer, is the most common malignant tumor and is the leading cause of cancer-related morbidity and mortality worldwide^1^. Non-small-cell lung carcinoma (NSCLC), including lung squamous cell carcinoma and lung adenocarcinoma (LUAD), accounts for approximately 85% of lung cancer^2^. Despite advances in clinical and experimental oncology, the early diagnostic and prognosis biomarkers of NSCLC is still unsatisfactory^3^. Therefore, there is an urgent need to identify new biomarkers to predict the clinical outcome of NSCLC patients.

Smoking, exposure to environmental tobacco smoking, residential radon, cooking oil fumes and particulate matter 2.5 (PM2.5) are main environmental risk factors associated to the occurrence of lung cancer^4,5^. Smoking is the major risk factor for lung cancer, accounting for about 90% of male and 70% of female^6^. Smoking causes multiple alterations to cells and tissues, including DNA single-strand breaks^7^, chromosome exchanges^8^ and chromosome instability (CIN)^9^, which can lead to genomic instability (GI). GI, mainly including CIN and microsatellite instability (MSI), has been an important hallmark of various human cancers^10–12^. GI has been proven to be closely related to the clinical diagnosis and prognosis of multiple malignant tumors^12,13^. Recent studies have clarified that GI can be used as a prognosis marker in cervical cancer^14^ and breast cancer^15^. Emerging evidence revealed that GI was associated with tumorigenesis in lung cancer^16,17^, however, the potential role and mechanism of GI in lung cancer need further be explored.

LncRNAs are defined as nonprotein coding transcripts more than 200 nucleotides in length^18^. Accumulating evidence suggested lncRNAs involve in gene expression at epigenetic, transcription and post-transcriptional levels^19–21^. Non-coding RNA activated by DNA damage (NORAD) and long non coding transcriptional activator of miR34a (GUARDIN) could maintain genomic stability by involving in DNA replication and repair in LUAD and colon cancer^22,23^. LncRNA DDSR1, a DNA damage-sensitive RNA1, modulated DNA repair by regulating homologous recombination in osteosarcoma^24^. However, the function and clinical roles of GI-associated lncRNAs in NSCLC remain unknown.

In this study, we systematically analyzed genomic data in LUAD from TCGA, and 185 differentially expressed GI-associated lncRNAs were enriched in chromosome formation and cell cycle checkpoint pathways. 5 GI-associated lncRNAs were identified through univariate and multivariate Cox regression analysis and used to construct a GI-associated lncRNAs signature (GILncSig) model. Our results demonstrated that the GILncSig may be a potential biomarker for the diagnosis and prognosis of LUAD and targeting 5 GI-associated lncRNAs could act as a therapeutic alternative for NSCLC.

## Materials and method

### Data acquisition

RNA sequencing transcriptome date (n = 594, Table S1), somatic mutation information (n = 561, Tables S2–3) and the corresponding clinicopathological features (n = 522, Table S4) of the LUAD patients were downloaded from TCGA (https://portal.gdc.cancer.gov/). The mutation information mainly refers to single-nucleotide variants (SNVs), copy number variation (CNV), MSI. LncRNA expression data were looked up from the previous analysed RNA expression data which were annotated by the GENCODE project (Version GRCh37, http://www.gencodegenes.org).

Samples from patients with overall survival (OS) of <  = 30 days were excluded^25^. In the end, a total of 490 LUAD patients with paired lncRNA and mRNA expression data, somatic mutation information and clinicopathological characteristics were enrolled in our study to build the GILncSig model.

**Figure 1:**
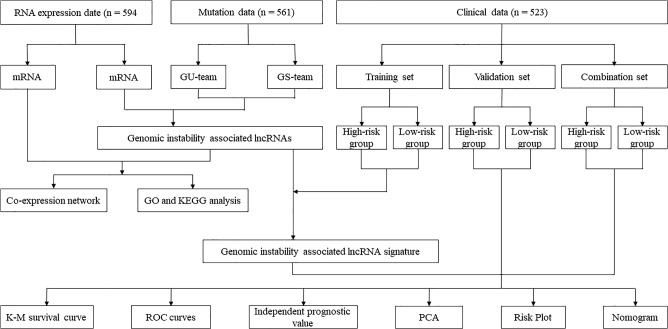
The flowchart depicting the process of data collection and analysis.

To increase the reliability of our research, we randomly and equally divided the entire dataset into a training set (n = 246), a validation set (n = 244) and the whole dataset was considered as a combination set (n = 490). The workflow of this work was shown in Fig. 1. Clinicopathological features of the 490 LUAD patients were shown in Table 1.

**Table 1:** Clinicopathological features of lung adenocarcinoma patients in the training set, validation set and combination set.

Characteristics		Training set (n = 246)	Validation set (n = 244)	Combination set (n = 490)	p value
Age	< = 65	117 (47.56%)	114 (46.72%)	231 (47.14%)	0.9945
	> 65	125 (50.81%)	124 (50.82%)	249 (50.82%)	
	Unknown	4 (1.63%)	6 (2.46%)	10 (2.04%)	
Sex	Female	131 (53.25%)	131 (53.69%)	262 (53.47%)	0.9950
	Male	115 (46.75%)	113 (46.31%)	228 (46.53%)	
Stage	Stage I–II	196 (79.67%)	182 (74.59%)	378 (77.14%)	0.2056
	Stage III–IV	46 (18.7%)	58 (23.77%)	104 (21.22%)	
	Unknown	4 (1.63%)	4 (1.64%)	8 (1.63%)	
T	T1–2	214 (86.99%)	212 (86.89%)	426 (86.94%)	0.9863
	T3–4	30 (12.2%)	31 (12.7%)	61 (12.45%)	
	Unknown	2 (0.81%)	1 (0.41%)	3 (0.61%)	
N	N0	164 (66.67%)	153 (62.7%)	317 (64.69%)	0.3671
	N1–3	76 (30.89%)	86 (35.25%)	162 (33.06%)	
	Unknown	6 (2.44%)	5 (2.05%)	11 (2.24%)	
M	M0	160 (65.04%)	164 (67.21%)	324 (66.12%)	1.0000
	M1	12 (4.88%)	12 (4.92%)	24 (4.9%)	
	Unknown	74 (30.08%)	68 (27.87%)	142 (28.98%)	

### Identification of GI-associated lncRNAs

To obtain GI-associated lncRNAs, as the method described by Bao et al.^26^: (a) the cumulative number of somatic mutations for each patient was calculated; (b) patients were arranged in a decreasing order based on their cumulative number of somatic mutations; (c) those in the top 25% of patients were categorized as genomic unstable (GU)-like team, while those in the last 25% were classified as genomically stable (GS)-like team; (d) expression profiles of lncRNAs between the GU-like team and GS-like team were compared using significance analysis of microarrays (SAM) method; (e) differentially expressed lncRNAs (log|fold change|> 1 and false discovery rate (FDR) adjusted P < 0.05) were defined as genome instability-associated lncRNAs.

### Evaluation of risk score

By linearly combining the expression value of GI-associated lncRNAs weighted by their coefficients, a risk-score formula was constructed as following^27^:

$${\text{Risk }}\;{\text{score }}\; = \;\mathop \sum \limits_{{\text{i = 1}}}^{{\text{n}}} {\text{coefi}}\; \times \;{\text{expri}}$$

where risk score was a prognostic risk score for the LUAD patients, coef_i_ represented the coefficient, and expr_i_ represented the expression of each prognostic GI-associated lncRNA. Based on the median risk score, the LUAD patients were classified into high-risk (n = 254) group and low-risk group (n = 236).

### Co-expression network

To measure the correlation between lncRNAs and mRNAs, Pearson correlation coefficients was conducted and the top 10 mRNAs were considered as co-expressed lncRNA-associated partners, a lncRNAs–mRNAs co-expression network was constructed.

### Functional enrichment analysis

To reveal the potential function of the co-expressed lncRNAs and mRNAs, Gene Ontology (GO) and Kyoto Encyclopedia of Genes and Genomes (KEGG) functional enrichment analysis of lncRNA-correlated PCGs were performed using clusterProfiler soft-ware.

### Building and validation of a Nomogram

To know the prognosis value of lncRNA, as the method employed by Iasonos et al.^28^, a nomogram was constructed by including the expression of the 5 lncRNAs in the combination set and a total score of 1-year, 2-year, and 3-year OS were evaluated. Calibration plot was further performed to evaluate the calibration and the discrimination of the nomogram by a bootstrap method with 1000 resamples.

### Statistical analysis

Hierarchical cluster analysis was performed using Euclidean distances and Ward’s linkage method. Kaplan–Meier analysis was used to calculate the OS. Univariate Cox and Multivariate Cox regression and stratified analysis were used to verify the independence of the GILncSig from other clinical factors and investigate the time-dependent prognostic value of the GILncSig in cancers. Hazard ratio (HR) and 95% confidence interval (CI) were calculated by Cox analysis. Receiver operating characteristic (ROC) was used to investigate the time-dependent prognostic value of the GILncSig. Principal component analysis (PCA) was performed to study the expression patterns in the different groups. All statistical analyses were performed using R-version 4.0.3. A P-value of less than 0.05 was considered statistically significant.

## Results

### Identification of GI-related lncRNAs in LUAD patients

**Figure 2:**
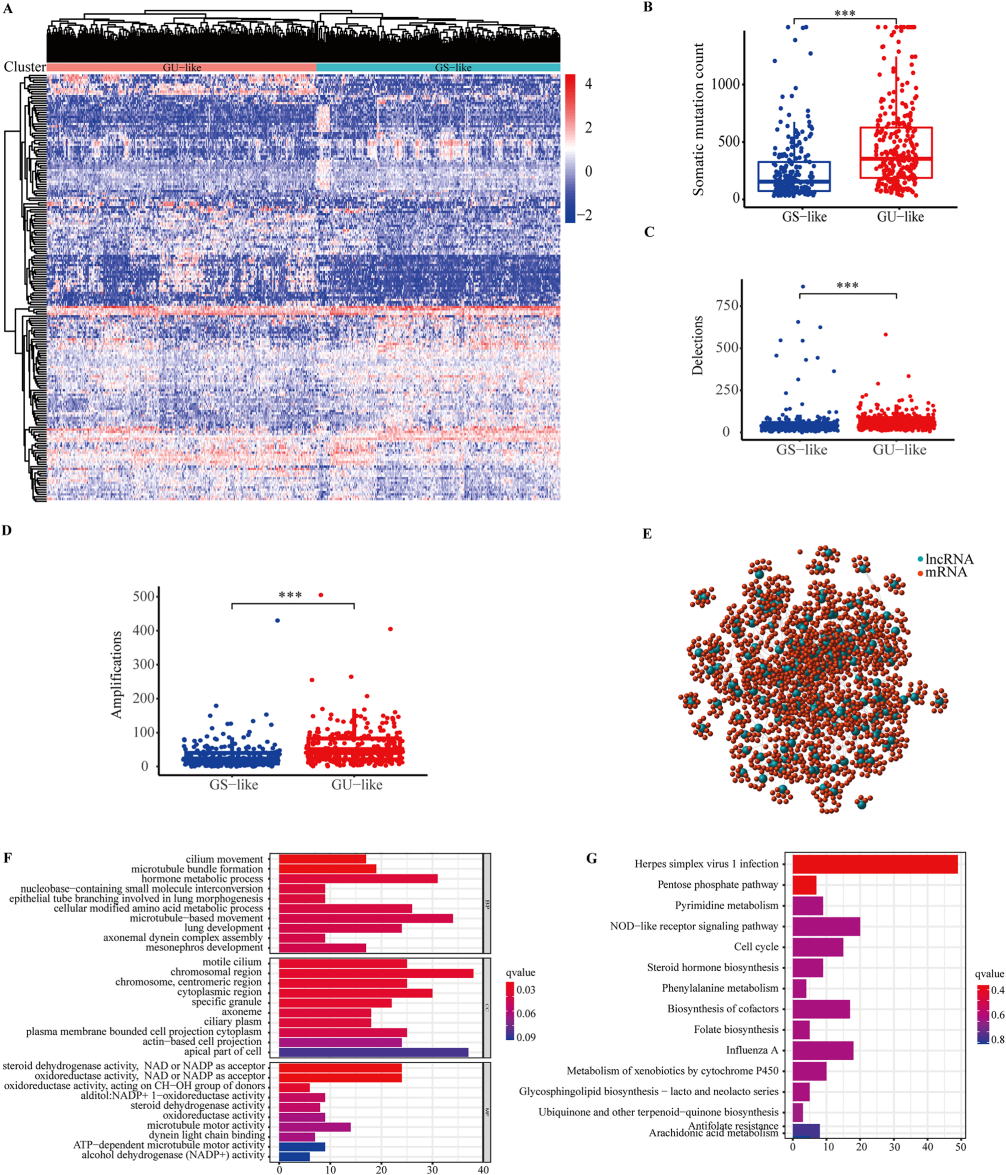
The identification of long non-coding RNAs related to GI and subsequent functional enrichment analysis. (**A**) Clustering analysis of 535 LUAD patients based on the expression of 185 candidate genomic instability-related lncRNAs. The left cluster is GU-like group, and the right cluster is GS-like group. The x-axis represents lung adenocarcinoma patients. The y-axis represents 185 candidate genomic instability-related lncRNAs. (**B**–**D**) Boxplots of somatic mutations (**B**), deletion mutation (**C**) and gene amplifications (**D**) in the GU-like group and GS-like group. (**E**) Co-expression network of GI-related lncRNAs and mRNAs based on the Pearson correlation coefficient. The blue circles represent lncRNAs, and the blue circles represent mRNAs. (**F**, **G**) Functional enrichment analysis of GO (**F**) and KEGG (**G**) for co-expressed lncRNAs and mRNAs. ***p < 0.001; LUAD, lung adenocarcinoma; GI, genomic instability; GU, genetic unstable; GS, genetic stable; GO, Gene Ontology; KEGG, Kyoto Encyclopedia of Genes and Genomes.

The cumulative number of somatic mutations in each patient was calculated and ranked to identify GI-associated lncRNAs. The first 25% of patients (n = 134) were assigned to GU-like team and the last 25% to GS-like team (n = 139) (Table S5). To find lncRNAs with significant differences, mRNA and lncRNA expression profiles (Tables S6–7) in each team were compared. With log|fold change|> 1 and FDR-adjusted P-value < 0.05, a total of 185 lncRNAs (candidate genomic instability-related lncRNAs) were considered to be significantly differentially expressed (Table S8). Hierarchical clustering analysis was conducted on the 535 samples in the TCGA set. Through the expression of the 185 differentially expressed lncRNAs, all 535 samples were clustered into two groups (Fig. 2A and Table S9). The group with higher cumulative somatic mutations was defined as GU-like group and the other GS-like group. The count of somatic cumulative mutations, deletion mutation and gene amplifications in the GU-like group was significantly higher than that in the GS-like group (p < 0.001, Fig. 2B–D).

To further explore the potential functions and pathways of the above 185 lncRNAs in LUAD, functional enrichment analysis was analyzed. The 10 most relevant protein-coding genes (PCGs) for each lncRNA among the 185 lncRNAs was found by using the method of co-expression analysis, then the lncRNAs–mRNAs co-expression network was constructed. In the network, the nodes were lncRNAs and mRNAs, they linked together if they were related to each other (Fig. 2E). The regulating effects of lncRNAs and mRNAs in the network were characterized in Table S10, all lncRNAs and mRNAs had cis regulating effects. GO and KEGG analysis of lncRNA-correlated PCGs revealed that mRNAs in this network were significantly associated with GI such as cilium movement, microtubule bundle formation, motile cilium, chromosomal region, microtubule motor activity and cell cycle (Fig. 2F and G). These results suggested that the 185 differentially expressed lncRNAs affected GI through lncRNA-related PCGs regulatory network and they can be candidate biomarkers for GI-associated lncRNAs.

### Identification of a GILncSig for prognostic validation in the training set

**Figure 3:**
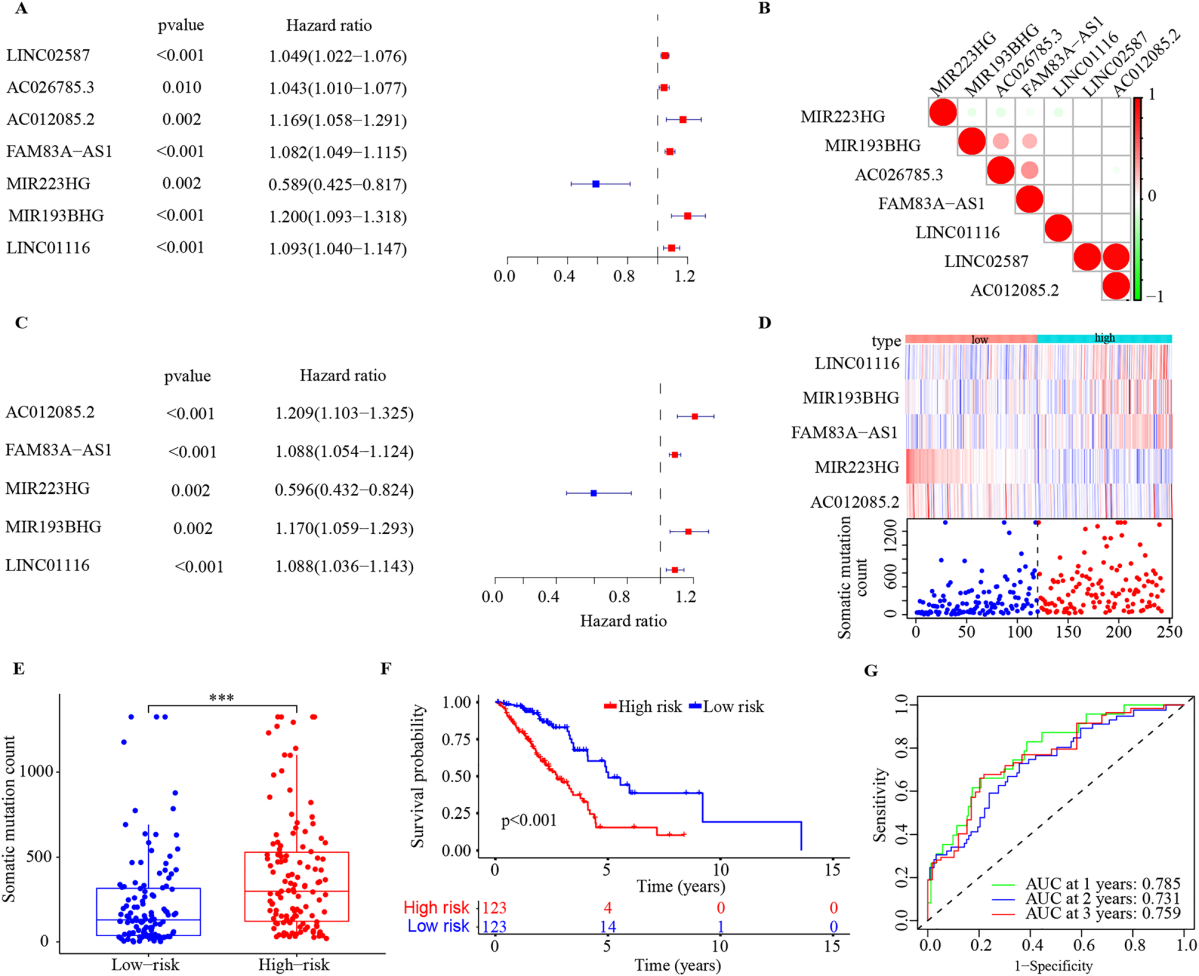
Identification of the GILncSig in the training set. (**A**) The forest plot of univariate cox regression identified 7 GI-associated lncRNAs. (**B**) The correlation analysis among the 7 GI-associated lncRNAs. (**C**) The forest plot of multivariate Cox regression analysis of 5 GI-associated lncRNAs. (**D**) The expression and somatic mutation count with increasing risk score of the GILncSig. (**E**) Boxplot of somatic mutation count in the high- and low-risk groups. (**F**) Kaplan–Meier survival curve of the high- and low-risk groups. (**G**) Time-dependent ROC curves and area AUC for 1-, 2-, and 3-year OS. *** p < 0.001; GI, genomic instability; ROC receiver operating characteristic; AUC, under the curve.

Univariate Cox regression analysis of the 185 differentially expressed lncRNAs in the training set showed that 7 GI-associated lncRNAs (LINC02587, AC026785.3, AC012085.2, FAM83A-AS1, MIR223HG, MIR193BHG, LINC01116) had the significant prognostic value in LUAD patients (p < 0.05, Fig. 3A). The correlation analysis indicated 7 GI-associated lncRNAs significantly interacted with each other (Fig. 3B). 5 GI-associated lncRNAs (AC012085.2, FAM83A-AS1, MIR223HG, MIR193BHG, LINC01116) were identified by multivariate Cox regression analysis and then were further used to develop the GILncSig model (Fig. 3C). In the GILncSig, AC012085.2, FAM83A-AS1, MIR193BHG, LINC01116 acted as risk factors for LUAD, and MIR223HG acted as a protective factor. The coefficients were shown in Table 2. The risk score of each patient was calculated by the sum of the coefficients of each lncRNA multiplied by the corresponding expression in each patient. Based on the median risk score, the training set was classified into high-risk group (n = 123) and low-risk group (n = 123). As shown in Fig. 3D, the expression level of the risk lncRNAs (AC012085.2, FAM83A-AS1, MIR193BHG, LINC01116) was upregulated, while the protective MIR223HG was downregulated, and the count of somatic mutations was positively correlated with the patient’s risk score. The count of somatic mutation of patients in the low-risk group was significantly lower than that of patients in the high-risk group (p < 0.001, Fig. 3E), and our results were similar to those of Matuno et al.^29^. The Kaplan–Meier curve indicated that the patients in the high-risk group had a poorer OS than those in the low-risk group (p < 0.001, Fig. 3F). The time-dependent ROC curve analysis of GILncSig for 1-, 2-, and 3-year OS were 0.785, 0.731 and 0.759 respectively (Fig. 3G). PCA analysis showed low- and high-risk groups were significantly distributed in two different directions, indicating that the LUAD patients in the low-risk group was quite distinguished from those in the high-risk group (Fig. S1A).

**Table 2:** 5 prognostic GI-associated lncRNAs identified from univariate and multivariate Cox regression analysis.

LncRNA	Coef	HR	HR.95L	HR.95H	p value
AC012085.2	0.1897	1.2089	1.1032	1.3246	4.81E−05
FAM83A-AS1	0.0847	1.0884	1.0542	1.1237	1.98E−07
MIR223HG	− 0.5170	0.5963	0.4317	0.8237	0.0017
MIR193BHG	0.1571	1.1701	1.0592	1.2926	0.0019
LINC01116	0.0845	1.0882	1.0360	1.1430	0.0007

### Validation of the GILncSig

**Figure 4:**
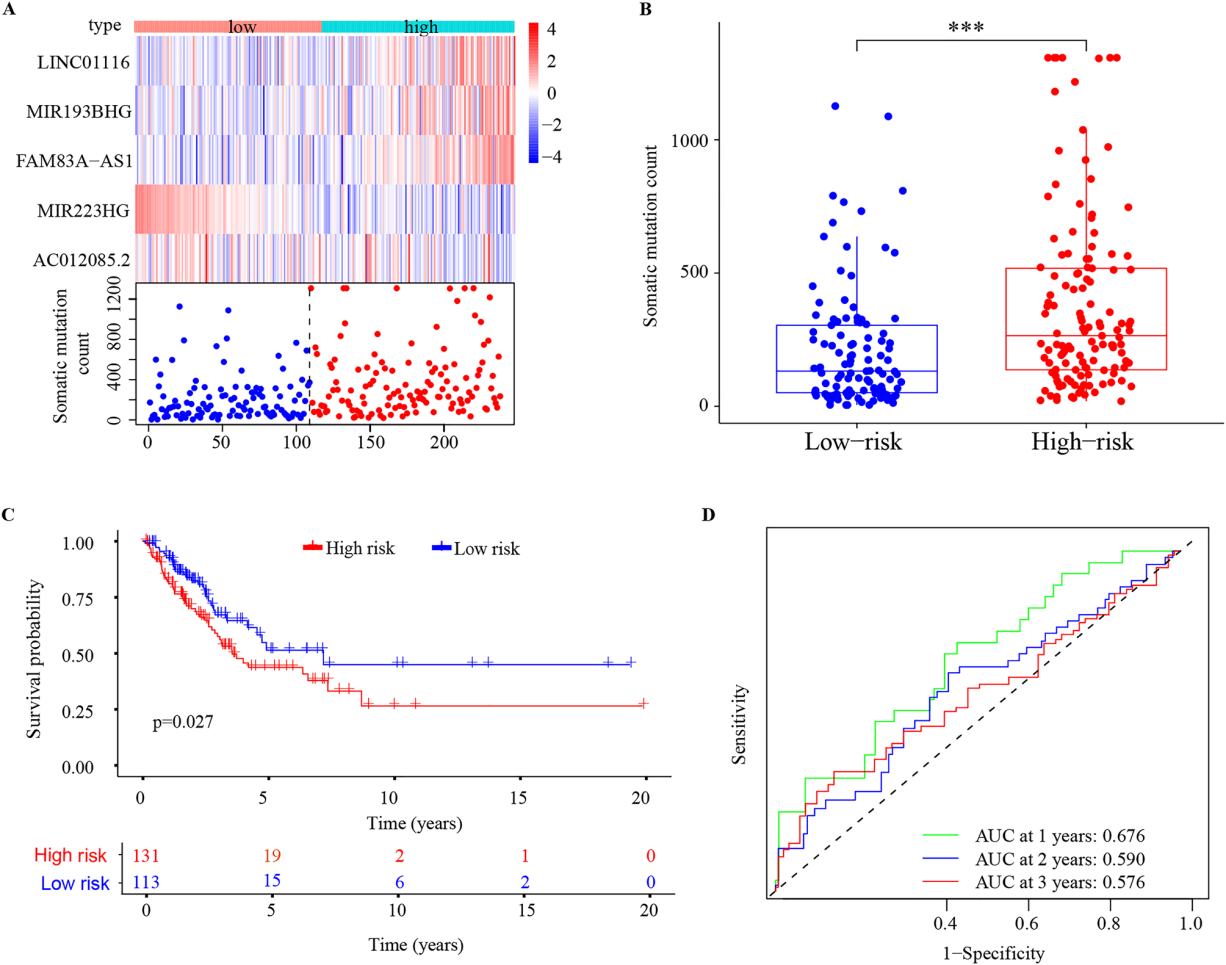
The prognostic values of the GILncSig in the validation set. (**A**) The expression and somatic mutation count with increasing risk score of the GILncSig. (**B**) Boxplot of somatic mutation count in the high- and low-risk groups. (**C**) Kaplan–Meier survival curve of the high- and low-risk groups. (**D**) ROC curves and AUC for 1-, 2-, and 3-year OS. ***p < 0.001; ROC receiver operating characteristic; AUC, under the curve; OS, overall survival.

**Figure 5:**
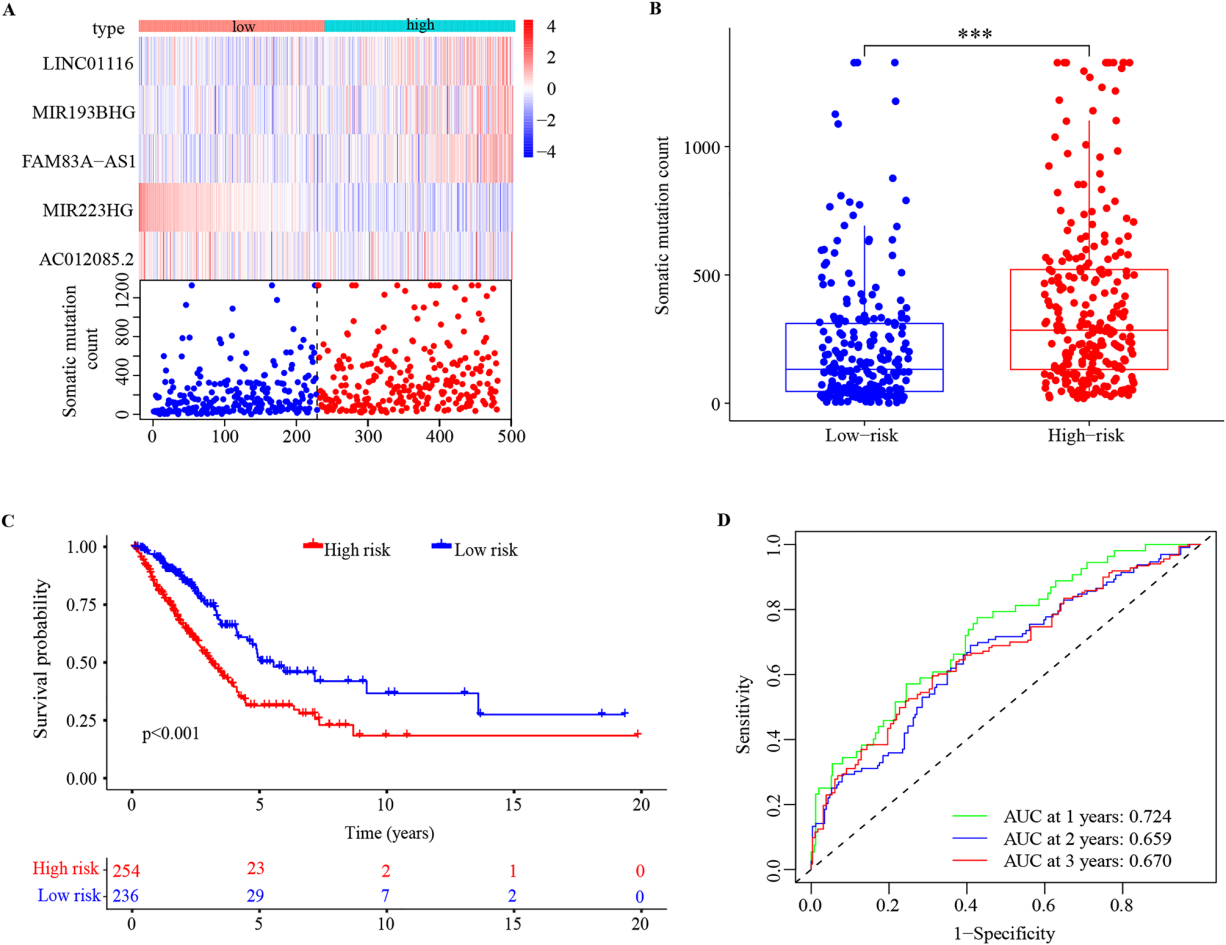
The prognostic values of the GILncSig in combination set. (**A**) The expression and somatic mutation count with increasing risk score of the GILncSig. (**B**) Boxplot of somatic mutation count in the high- and low-risk groups. (**C**) Kaplan–Meier survival curve of the high- and low-risk groups. (**D**) ROC curves and AUC for 1-, 2-, and 3-year OS. ***p < 0.001; ROC receiver operating characteristic; AUC, under the curve; OS, overall survival.

To further verify the accuracy of the GILncSig model, the 5 coefficients were applied to the validation set (n = 244) and the combination set (n = 490) to confirm the risk score of each patient, then the validation set was classified into the high-risk group (n = 131) and low-risk group (n = 113) and the combination set was classified into the high-risk group (n = 254) and low-risk group (n = 235). In the validation set, with the increase of patient’s risk score, the expression level of the risk lncRNAs was also upregulated, the protective lncRNAs was downregulated and the count of somatic mutations also increased (Fig. 4A). The somatic mutation count of patients in the low-risk group was lower than that of patients in the high-risk group (p < 0.001, Fig. 4B). Patients in the low-risk group had better OS rates than in the high-risk group (p = 0.027, Fig. 4C). The ROC curves showed that the AUCs for the 1-, 2-, and 3-year OS were 0.676, 0.590, and 0.576 (Fig. 4D). PCA showed that the low- and high-risk groups were divided into two different clusters (Fig. S1B). Similarly, the results were also validated in the combination set (p < 0.001, Figs. 5A–D and S1C).

Univariate Cox regression analysis was performed using the survival package to investigate the time-dependent prognostic value of the GILncSig in cancers. The results indicate that the GILncSig is a significant risk factor for Adrenocortical carcinoma (ACC), Bladder Urothelial Carcinoma (BLCA), Colon adenocarcinoma (COAD), Kidney renal papillary cell carcinoma (KIRP), Brain Lower Grade Glioma (LGG), LUAD, Pancreatic adenocarcinoma (PAAD), Stomach adenocarcinoma (STAD) and Thymoma (THYM) (Fig. S2 and Table S11).

### Somatic mutation types in the combination set

To explore the mutation types of LUAD, the R package “maftools” was used to visualize mutation data from 490 samples in TCGA-LUAD. In the high-risk group, missense mutation was predominant in variant classification (Fig. S3A and S3E); SNP was the most frequent in variant type (Fig. S3B); and the mutation from C to A was the most prevalent in the SNV class (Fig. S3C). Additionally, the median number of variants per sample was 226 (Fig. S3D). The top 10 mutated genes included TTN, MUC16, CSMD3, RYR2, TP53, LRP1B, ZFHX4, USH2A, FLG, and KRAS (Fig. S3F). Similar results were also found in the low-risk group (Fig.S3G–L). The low-risk group had significant higher arm-level amplification and deletion frequencies than the low-risk group (P < 0.05) in the combination set (Fig. S3M). In conclusion, we observed a distinct pattern in the occurrence of mutations during the progression of LUAD.

### Independence of the GILncSig from other clinical factors

To identify the independent prognostic value of GILncSig, univariate and multivariate Cox regression analysis were performed on age, sex, TNM stage, and the risk signature. The tumor TNM stage is determined based on the size of the tumor (Tumor), involvement of lymph nodes (Node), and presence of metastasis (Metastasis) to assess the severity and prognosis of the tumor. The results indicated that the risk signature and TNM stage were independent factors when adjusted for age, sex and smoking in all sets (Table 3).

**Table 3:** Univariate and multivariate Cox regression analysis of the GILncSig and overall survival in each set.

Variables	Univariate Cox regression analysis				Multivariate Cox regression analysis			
	HR	HR.95L	HR.95H	p value	HR	HR.95L	HR.95H	p value
Training set								
Age	0.9875	0.9661	1.0092	0.2570	0.9989	0.9768	1.0214	0.9220
Sex	0.9769	0.6410	1.4889	0.9134	0.9823	0.6391	1.5099	0.9352
TNM stage	1.8486	1.4888	2.2955	**2.66E-08**	1.7412	1.3860	2.1874	**1.89E−06**
Risk score	1.0494	1.0291	1.0702	**1.39E−06**	1.0370	1.0160	1.0582	**0.0005**
Validation set								
Age	1.0168	0.9939	1.0401	0.1526	1.0169	0.9946	1.0397	0.1384
Sex	1.3072	0.8489	2.0129	0.2238	1.0383	0.6622	1.6281	0.8699
TNM stage	1.6099	1.3242	1.9572	**1.78E−06**	1.6050	1.3121	1.9634	**4.18E−06**
Risk score	1.0525	1.0132	1.0933	**0.0083**	1.0521	1.0100	1.0960	**0.0145**
Combination set								
Age	1.0047	0.9893	1.0202	0.5528	1.0101	0.9949	1.0257	0.1948
Sex	1.1126	0.8251	1.5003	0.4841	0.9958	0.7343	1.3504	0.9784
TNM stage	1.6412	1.4254	1.8897	**5.73E−12**	1.6234	1.4048	1.8761	**5.20E−11**
Risk score	1.0187	1.0105	1.0270	**6.39E−06**	1.0163	1.0085	1.0243	**4.26E−05**

Significant values are in [bold].

**Figure 6:**
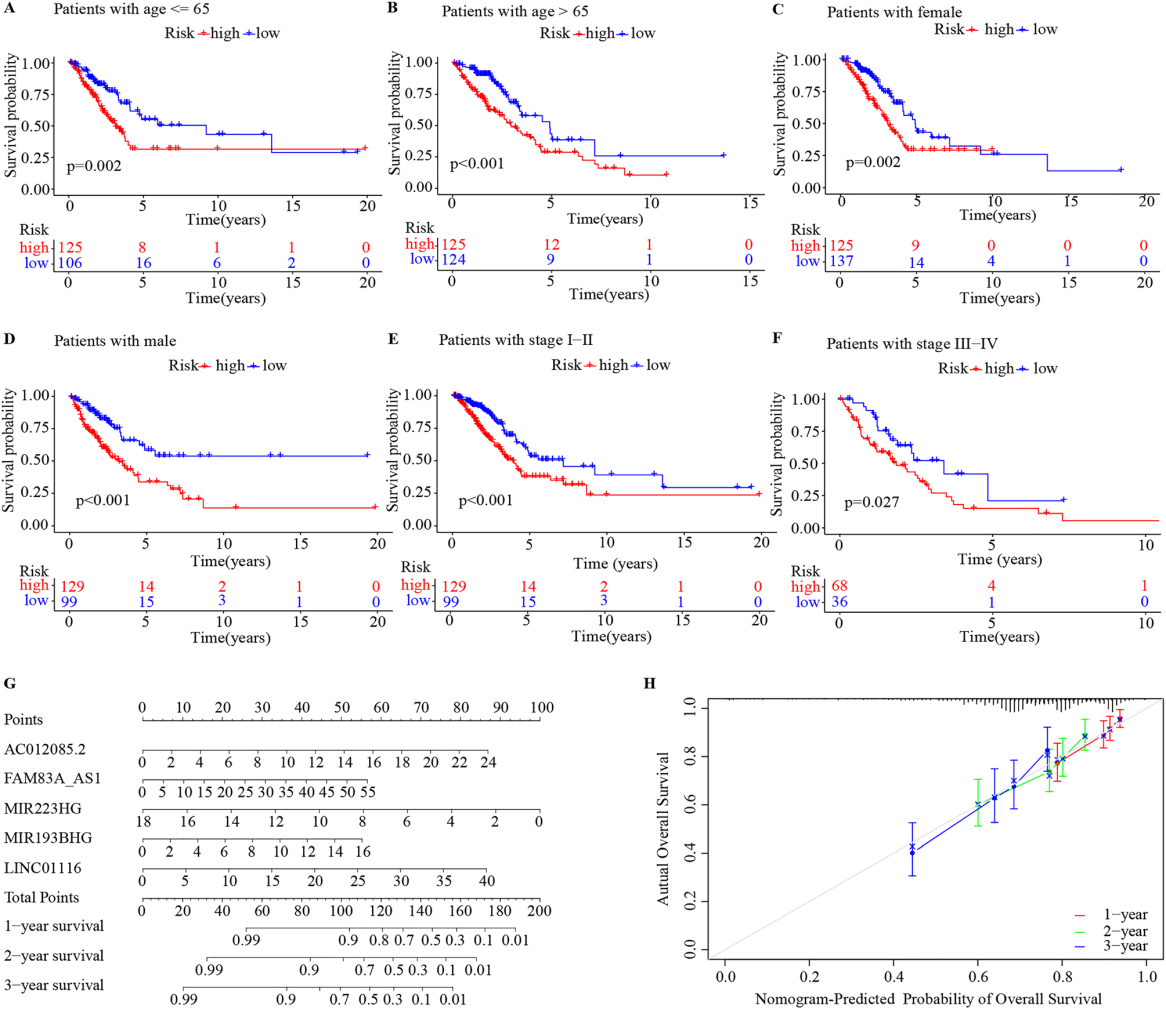
Stratified survival analyses and nomogram of the GILncSig in the combination set. (**A**–**F**) Kaplan–Meier survival curves in subgroups stratified by different clinical characteristics. Age <  = 65 (**A**), Age > 65 (**B**), Female (**C**), Male (**D**), Stage I + II (**E**), Stage III + IV (**F**). (**G**) Nomogram for predicting 1-, 2-, and 3-year OS of LUAD patients. (**H**) Calibration curves for the nomogram. OS, overall survival; LUAD, lung adenocarcinoma.

In the multivariate Cox regression analysis, TNM stage were also identified as independent prognostic factor. Subsequently, a stratification analysis was performed to evaluate whether the GILncSig could predict patient survival within the same clinical factor subgroup. Patients in the combination group were stratified based on clinical parameters, such as age (< = 65/ > 65), sex (female/male), stage (I + II/III + IV). The results showed that the GILncSig could classify patients of the same stratum of age, sex, and stage into high- and low-risk groups. Patients in the high-risk group had a poorer OS than those with low-risk group in each stratum (Fig. 6A–F. These results indicated that the GILncSig was an independent prognostic factor related to the OS in LUAD.

### Construction of a nomogram based on the GILncSig in the combination set

To construct a quantitative method for the prognosis of LUAD patients, we integrated the 5 GI-associated lncRNAs to establish a nomogram (Fig. 6G). The calibration curve for the nomogram indicated that using the nomogram to predict OS was highly consistent with actual OS (Fig. 6H).

## Discussion

Since lung cancer has no symptoms in the early stage, majority of patients are already in the advanced stage when they are discovered. Although traditional treatments are constantly improving, the five-year survival rate of lung cancer is only 19%^30^. GI has been reported in various malignant cancers, including lung cancers^11,14,16,17,31–35^. CIN is the major type of GI in lung cancer, which leads to the high gene mutation burden by chromosome structure and number alterations in cancer cells^11^. In this study, we found that a total of 185 GI-associated lncRNAs were significantly differentially expressed. Functional analysis revealed that GI-associated pathways were significantly enriched, which indicates that GI-associated lncRNAs may be associated with tumorigenesis. 5 GI-associated lncRNAs significantly associated with OS. A novel prognostic model integrating 5 GI-associated lncRNAs was firstly constructed and differentiate different risk group.

Among the 5 GI-associated lncRNAs, 4 of them, including AC012085.2, FAM83A-AS1, MIR193BHG, LINC01116, acted as risk factors for LUAD, and MIR223HG was a protective factor. FAM83A-AS1 was up-regulated in lung cancer tissues and enhances the proliferation, migration, invasion, and epithelial-mesenchymal transition of LUAD^36–38^. IR193BHG was elevated and showed good clinical values for diagnosing early-onset preeclampsia^39^. LINC01116 mediated gefitinib resistance of NSCLC cells by affecting IFI44 expression^40^. LINC01116 overexpressed in lung cancer tissues and cell lines and was significantly associated with proliferation and metastasis^40–42^. MIR223HG, acting as a competing endogenous RNA, inhibited acute myeloid leukemia progression by inducing IRF4 expression^43^. These studies were consistent with our results. However, the role of AC012085.2 was unclear and need be further explored.

KEGG and GO enrichment analysis indicated that genes co-expressed with the 185 GI-related lncRNAs in the high-risk and low-risk LUAD patients identified in this study were not only enriched in many biological processes, such as metabolic process and oxidoreductase activity, they were also enriched in critical GI-related pathways, including cilium movement pathway, microtubule motor activity pathway, chromosomal region pathway, and cell cycle pathway. Dyskinesia and structural abnormalities of cilia can cause GI and lead to the occurrence of cancer^44^. Chromosome segregation requires stable microtubule attachment at kinetochores, when the dynamic of microtubules is insufficient and the orientation is deviated, it can lead to GI in human cells^45,46^. The mechanisms of GI also include extra centrosomes^47^, mutations of mitotic checkpoint genes^48^, faulty cell-cycle regulation^49–51^, and chromatid cohesion^52^. The mechanism of GI is very complicated and needs be further studied.

Meanwhile, there are several limitations to our study. (1) Due to the limited lncRNA chip of LUAD, we could only divide the TCGA data set into training set and validation set randomly and more independent data sets are needed to validate the GILncSig to ensure its robustness and reproducibility. (2) In vivo and in vitro experiments were not performed to verify the role of the GILncSig model, therefore, subsequent experiments are needed to validate the reliability of results. (3) As an observational study, confounding factors, such as pharmaceutical treatment, exposure to environmental tobacco smoking, residential radon, cooking oil fumes, PM2.5 and so on, may have an impact on our results.

## Conclusions

In summary, in this study, we constructed a novel GILncSig model that may involve in the progression and prognosis in LUAD. Targeting GI-related lncRNAs may be a potential application for LUAD therapy. However, the underlying mechanisms involving in GI need be further explored.

### Supplementary Information

Supplementary Figure S1.

Supplementary Figure S2.

Supplementary Figure S3.

Supplementary Legends.

Supplementary Table S1.

Supplementary Table S2.

Supplementary Table S3.

Supplementary Table S4.

Supplementary Table S5.

Supplementary Table S6.

Supplementary Table S7.

Supplementary Table S8.

Supplementary Table S9.

Supplementary Table S10.

Supplementary Table S11.

## Data Availability

RNA sequencing date, somatic mutation information and the clinicopathological features used and analyzed during the current study are available from TCGA (https://portal.gdc.cancer.gov/).

## Abbreviations

GI: Genomic instability
lncRNA: Long non-coding RNA
GILncSig: GI-related lncRNA signature
LUAD: Lung adenocarcinoma
TCGA: The Cancer Genome Atlas
OS: Overall survival
NSCLC: Non-small-cell lung carcinoma
CIN: Chromosome instability
MSI: Microsatellite instability
NORAD: Non-coding RNA activated by DNA damage
GUARDIN: Long non coding transcriptional activator of miR34a
DDSR1: A DNA damage-sensitive RNA1
SNVs: Single-nucleotide variants
CNV: Copy number variation
GU: Genetic unstable
GS: Genetic stable
SAM: Significance analysis of microarrays
FDR: False discovery rate (FDR)
GO: Gene Ontology
KEGG: Kyoto Encyclopedia of Genes and Genomes
HR: Hazard ratio
CI: Confidence interval
ROC: Receiver operating characteristic
PCA: Principal component analysis
PCGs: Protein-coding genes
AUC: Under the curve
ACC: Adrenocortical carcinoma
BLCA: Bladder Urothelial Carcinoma
COAD: Colon adenocarcinoma
KIRP: Kidney renal papillary cell carcinoma
LGG: Brain Lower Grade Glioma
PAAD: Pancreatic adenocarcinoma
STAD: Stomach adenocarcinoma
THYM: Thymoma
KIRC: Kidney Chromophobe
HNSC: Head and Neck squamous cell carcinoma
CECS: Cervical squamous cell carcinoma and endocervical adenocarcinoma
PCPG: Pheochromocytoma and Paraganglioma
BRCA: Breast invasive carcinoma
OV: Ovarian serous cystadenocarcinoma
SKCM: Skin Cutaneous Melanoma
SARC: Sarcoma
ESCA: Esophageal carcinoma
MESO: Mesothelioma
UCS: Uterine Carcinosarcoma
LUSC: Lung squamous cell carcinoma
GBM: Glioblastoma multiforme
KICH: Kidney Chromophobe
LIHC: Liver hepatocellular carcinoma
UCEC: Uterine Corpus Endometrial Carcinoma
CHOL: Cholangio carcinoma
TGCT: Testicular Germ Cell Tumors
READ: Rectum adenocarcinoma
THCA: Thyroid carcinoma
DLBC: Lymphoid Neoplasm Diffuse Large B-cell Lymphoma
PRAD: Prostate adenocarcinoma
UVM: Uveal Melanoma
DSS: Disease-specific survival
OS: Overall survival
PFI: Progression-free interval
