# Supplementary figures and images for: A genomic instability-associated lncRNA signature for predicting prognosis and biomarkers in lung adenocarcinoma
Source: Sci Rep. 2024 Jun 24;14:14460. doi: 10.1038/s41598-024-65327-3 (PMC11196711; doi:10.1038/s41598-024-65327-3)

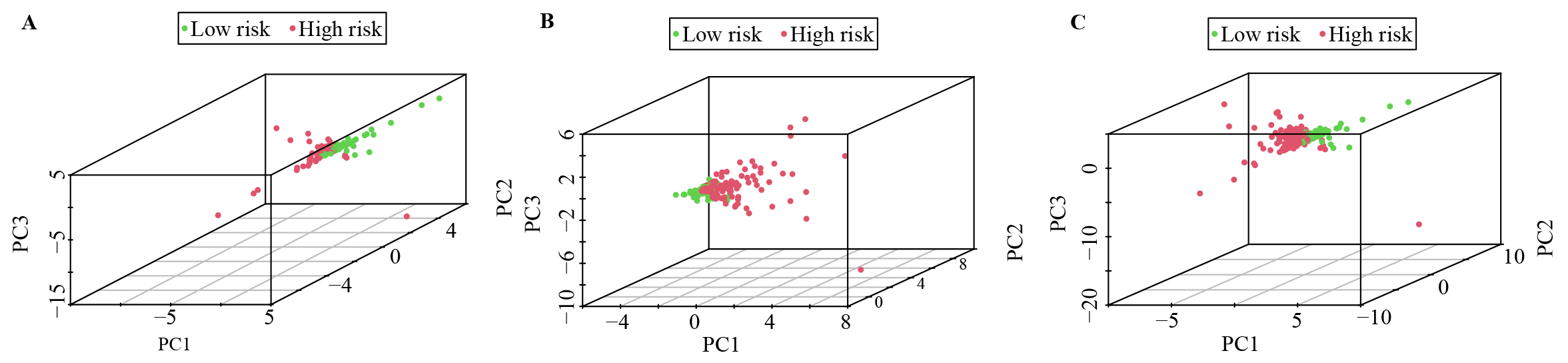

Supplement: Supplementary file 1 — Supplementary Figure S1. [file 41598_2024_65327_MOESM1_ESM.tif]

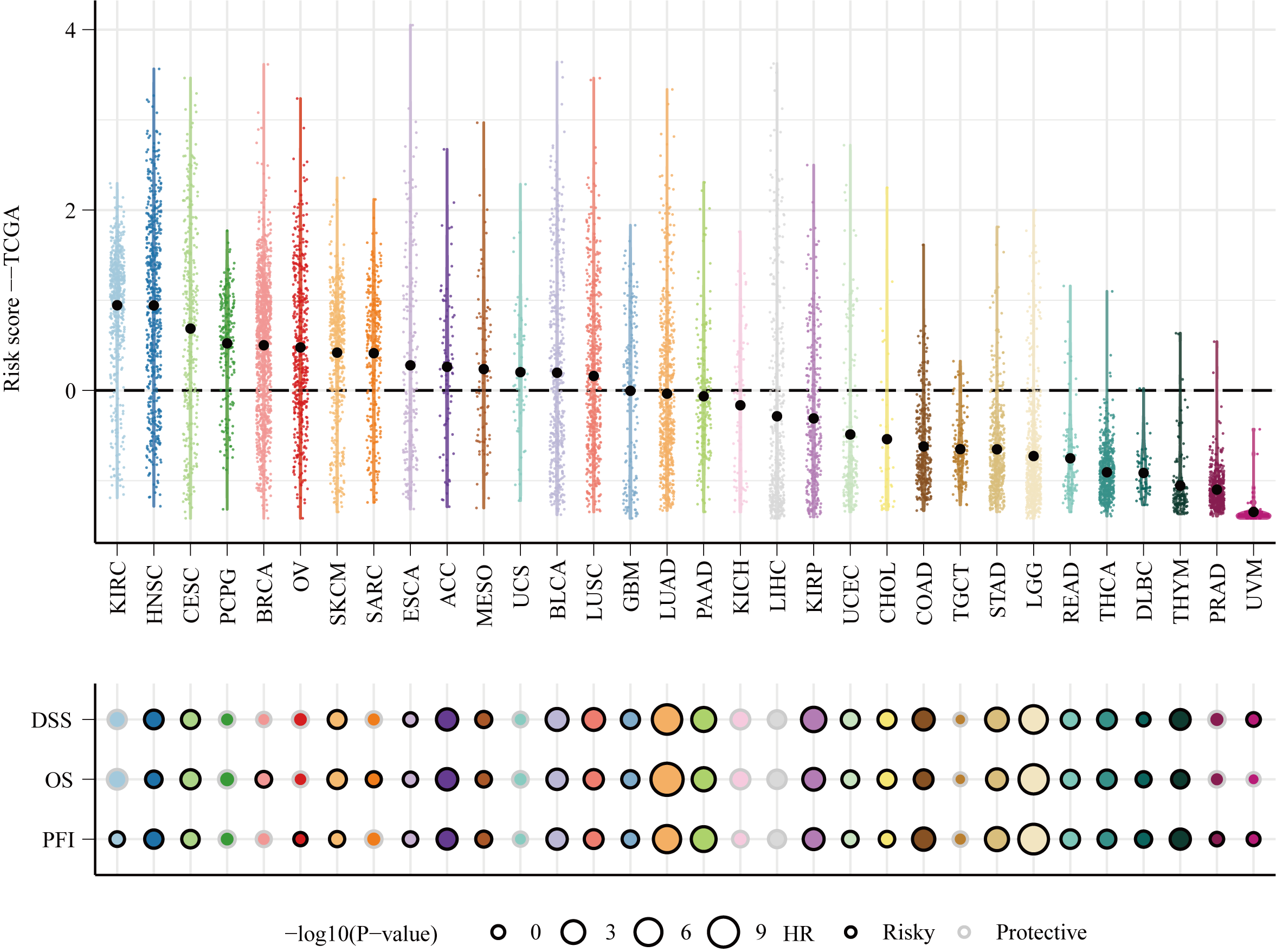

Supplement: Supplementary file 2 — Supplementary Figure S2. [file 41598_2024_65327_MOESM2_ESM.tif]

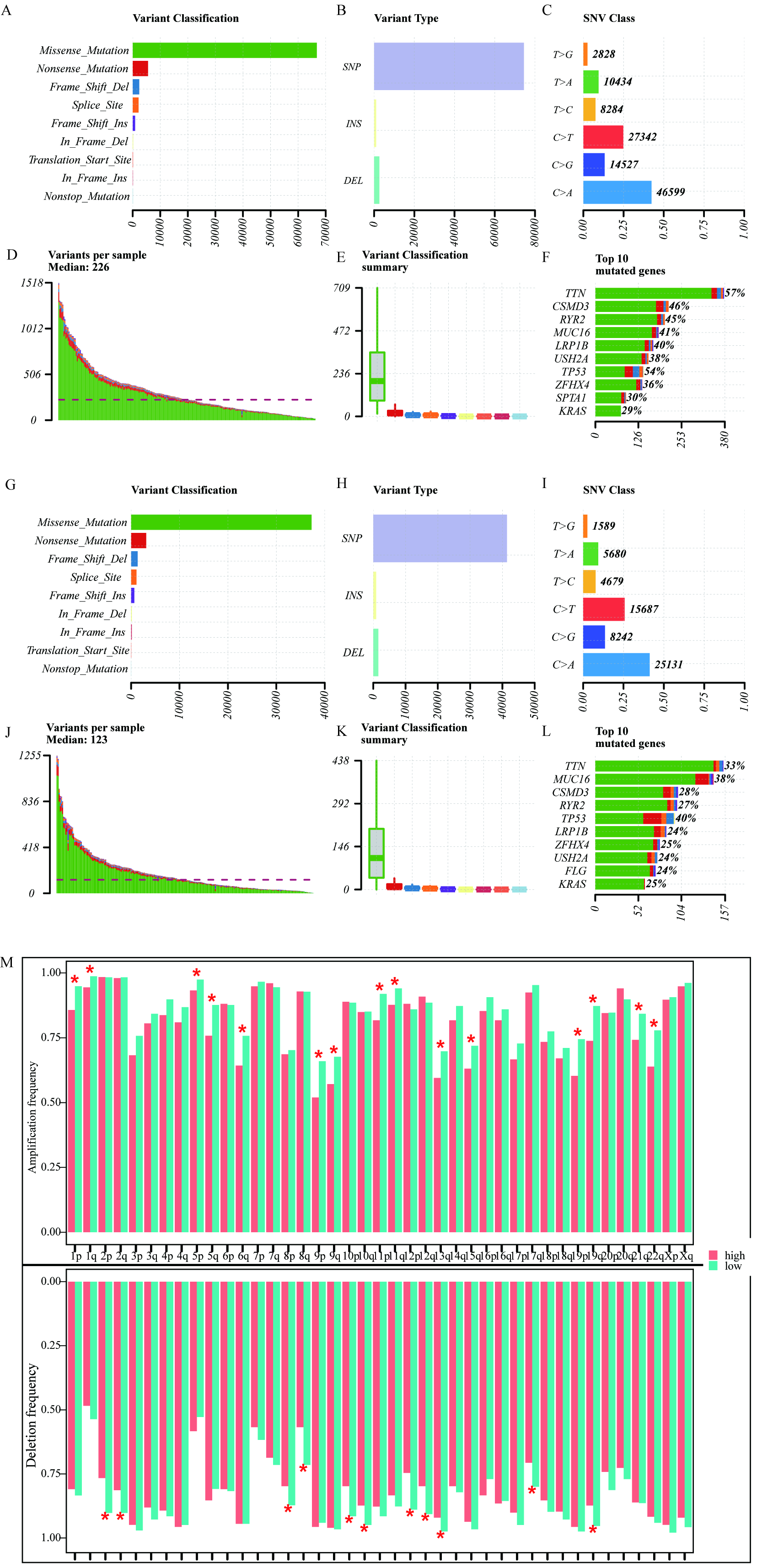

Supplement: Supplementary file 3 — Supplementary Figure S3. [file 41598_2024_65327_MOESM3_ESM.tif]
