# Supplementary material for: A genomic instability-associated lncRNA signature for predicting prognosis and biomarkers in lung adenocarcinoma
Source: Sci Rep. 2024 Jun 24;14:14460. doi: 10.1038/s41598-024-65327-3 (PMC11196711; doi:10.1038/s41598-024-65327-3)
Supplement: Supplementary file 4 — Supplementary Legends. [file 41598_2024_65327_MOESM4_ESM.docx]

**Figure S1.** Principal Component Analysis. (A-C) Principal Component Analysis based on the GILncSig indicated low-risk and high-risk groups were generally distributed in two different directions in the training set (A), validation set (B) and combination set (C).

**Figure S2**. **The application of the GILncSig in pan-cancer**. Univariate Cox results of DSS, OS and PFI in 33 human cancers. HR > 1 represents the risk factors for survival, represented by a black circle, and HR < 1 represents protective factors for survival, represented by a gray circle. The larger the circle, the more significant the difference. DSS, disease-specific survival; OS, overall survival; PFI, progression-free interval; HR, Hazard ratio; TCGA, The Cancer Genome Atlas; KIRC, Kidney Chromophobe; HNSC, Head and Neck   squamous cell carcinoma; CECS, Cervical squamous   cell carcinoma and endocervical adenocarcinoma; PCPG, Pheochromocytoma and   Paraganglioma; BRCA, Breast invasive carcinoma; OV, Ovarian serous cystadenocarcinoma; SKCM, Skin Cutaneous   Melanoma; SARC, Sarcoma; ESCA, Esophageal carcinoma; ACC, Adrenocortical   carcinoma; MESO, Mesothelioma; UCS, Uterine   Carcinosarcoma; BLCA, Bladder Urothelial   Carcinoma; LUSC, Lung squamous cell   carcinoma; GBM, Glioblastoma   multiforme; LUAD, Lung adenocarcinoma; PAAD, Pancreatic   adenocarcinoma; KICH, Kidney Chromophobe; LIHC, Liver hepatocellular   carcinoma; KIRP, Kidney renal   papillary cell carcinoma; UCEC, Uterine Corpus   Endometrial Carcinoma; CHOL, Cholangio carcinoma; COAD, Colon adenocarcinoma; TGCT, Testicular Germ Cell   Tumors; STAD, Stomach   adenocarcinoma; LGG, Brain Lower Grade Glioma; READ, Rectum adenocarcinoma; THCA, Thyroid carcinoma; DLBC, Lymphoid Neoplasm Diffuse Large B-cell Lymphoma; THYM, Thymoma; PRAD, Prostate   adenocarcinoma; UVM, Uveal Melanoma.

**Figure S3. Somatic mutation types in the combination set.** According to different classification methods in the high-risk group, variant classification (A), variant type (B), SNV class (C) were analyzed. (D) Histogram indicates the total number of variants per sample of LUAD. (E) Box plot shows the distribution of variant classification. (F) Top 10 mutated genes in LUAD. While in the low-risk group, the analysis results are similar (Figure S2G-L). (M) Comparisons of arm-level amplification and deletion frequencies between high- and low-risk groups. LUAD, Lung adenocarcinoma.
